# Expert consensus on protocol of rehabilitation for COVID‐19 patients using framework and approaches of WHO International Family Classifications

**DOI:** 10.1002/agm2.12120

**Published:** 2020-07-06

**Authors:** Bin Zeng, Di Chen, Zhuoying Qiu, Minsheng Zhang, Guoxiang Wang, Rehabilitation Group of Geriatric Medicine branch of Chinese Medical Association, division of Management of Medical Rehabilitation Institution of Chinese Hospital Association, Rehabilitation Institution Management division of Chinese Rehabilitation Medical Association, division of Rehabilitation Psychology, Chinese Psychological Association, division of Disability Classification Research, Chinese Association of Rehabilitation of Disabled Persons, Jianye Wang, Pulin Yu, Xianguang Wu, Bingchen An, Dingqun Bai, Zhuoming Chen, Jingyuan Deng, Qi Guo, Chengqi He, Xiquan Hu, Chongxia Huang, Qiuchen Huang, Xuming Huang, Zhen Huang, Xinping Li, Zhongming Liang, Gang Liu, Peng Liu, Chao Ma, Hongzhuo Ma, Zhongxiang Mi, Cuihuan Pan, Xiue Shi, Hongwei Sun, Jianing Xi, Xiaofei Xiao, Tao Xu, Wuhua Xu, Jian Yang, Shaohua Yang, Wanzhang Yang, Xiangming Ye, Xiaoping Yun, Aiming Zhang, Chong Zhang, Pande Zhang, Qiaojun Zhang, Mingming Zhao, Jiejiao Zhao

**Affiliations:** ^1^ Guangdong People’s Hospital Guangdong Academy of Medical Sciences Guangzhou China; ^2^ Division of Disability Classification Research of China Association of Rehabilitation of Disabled Persons Beijing China; ^3^ Research Institute of Rehabilitation Information China Rehabilitation Science Institute/China Rehabilitation Research Center Beijing China; ^4^ World Health Organization Collaborating Center of Family International Classifications in China Beijing China; ^5^ China Academy of ICF Weifang Medical University Weifang China; ^6^ Division of Rehabilitation Psychology Chinese Psychological Society Beijing China; ^7^ School of Physical Education and Sport Sciences/Institute of Exercise Rehabilitation Soochow University Jiangsu China; ^8^ WHO Collaborating Center of Family International Classifications in China Beijing China; ^9^ China Academy of ICF of Weifang Medical University Weifang, Shandong China; ^10^ Division of Disability Classification Research of China Association of Rehabilitation of Disabled Persons Beijing China

**Keywords:** COVID‐19, International Classification of Functioning, Disability and Health (ICF), rehabilitation

## Abstract

Coronavirus disease 2019 (COVID‐19) has widely spread all over the world and the numbers of patients and deaths are increasing. According to the epidemiology, virology, and clinical practice, there are varying degrees of changes in patients, involving the human body structure and function and the activity and participation. Based on the World Health Organization (WHO) International Classification of Functioning, Disability and Health (ICF) and its biopsychosocial model of functioning, we use the WHO Family of International Classifications (WHO‐FICs) framework to form an expert consensus on the COVID‐19 rehabilitation program, focusing on the diagnosis and evaluation of disease and functioning, and service delivery of rehabilitation, and to establish a standard rehabilitation framework, terminology system, and evaluation and intervention systems based the WHO‐FICs.

## 1 BACKGROUND

According to the World Health Organization (WHO), as of 10 am (utc+8 hours), April 24, 2020, coronavirus disease 2019 (COVID‐19) had been confirmed globally in 2 591 015 cases, resulting in 178 686 deaths (BBC). The “Novel Coronavirus Pneumonia Treatment and Treatment Plan (Trial Version 7)”^1^ issued by the China National Health and Health Committee reveals that in the acute stage, patients may present with increased respiratory secretions, multiple airway obstruction, intrapulmonary exudation, atelectasis and other respiratory system lesions, as well as necrosis, degeneration, tissue edema, and other pathological changes in multiple organs outside the lung. Elderly patients and severe and critical patients may have a variety of underlying diseases or may suffer from the direct invasion of multiple organ systems, resulting in different degrees of functioning, which may manifest as hypoxemia, decreased airway clearance ability, fatigue, incapacity, decreased muscle strength and endurance, activity limitation, and participation restriction, leading to a significant decline in quality of life.

Based on the WHO’s International Classification of Functioning, Disability and Health (ICF) and its biopsychosocial model of functioning and disability, and relevant assessments, we use the WHO Family International Classifications (WHO‐FICs)^2^, ^3^ framework and form the expert consensus on COVID‐19 rehabilitation programs to recommend a series of measures to maximize patients’ functioning and improve their quality of life.^4^, ^5^

This consensus focuses on a series of objectives as follows: (1) the establishment of a rehabilitation framework, terminology, and evaluation and intervention approach in accordance with WHO‐FICs standards; (2) the integration of rehabilitation services into the continuum of health services for prevention, treatment, rehabilitation, and health promotion in response to the rehabilitation needs of COVID‐19 patients; (3) the provision of multidisciplinary, interdisciplinary, and lifespan rehabilitation services to patients in medical institutions, rehabilitation institutions, and communities; (4) improvement of the quality and safety of rehabilitation services; (5) collection of big data on rehabilitation; and (6) the provision of an evidence‐based foundation for the establishment of clinical practice recommendations for the rehabilitation of COVID‐19 cases.^6^

## 2 METHODOLOGY AND EVIDENCE FOR EXPERT CONSENSUS

### 2.1 WHO Family International Classifications

The WHO‐FICs include three reference classifications: the International Classification of Diseases (ICD),^7^ the ICF,^8^, ^9^, ^10^, ^11^, ^12^ and the International Classification of Health Interventions (ICHI).^13^ The ICD is a classification system of diseases, injuries and causes. The latest version is the ICD‐11. We used ICD‐11 and ICD‐10 to develop diagnostic protocol of COVID‐19. The ICF has established a unified and standardized terminology system to classify the functioning and disability. It is the fundamental system of physical medicine and rehabilitation and is recommended in the fields of diagnosis and coding, evaluation, and interventions of functioning to maximize patients’ functioning at three levels: (1) body function and structure; (2) activity and participation; and (3) environmental factors and personal factors. The ICHI provides a set of general classifications to report and analyze the evaluation and health interventions. It is applicable to all health system levels and uses the same structure and terminology as the ICF to describe health interventions. The ICD is used for disease diagnosis and coding; the ICF is used for description, evaluation, and coding of functioning; and the ICHI is used for intervention and coding of functioning. The ICHI is consistent with the ICD‐11 and ICF in ontological structure and terminology.^14^, ^15^

[Consensus 1] This consensus adopts the framework and approach of WHO‐FICs to build a rehabilitation protocol of COVID‐19 disease diagnosis, description and evaluation, coding and intervention of functioning (see Table 1).

**TABLE 1:** Protocol of rehabilitation for COVID‐19 cases using the WHO‐FICs

Diagnosis and codes of COVID‐19 with ICD‐10 (http://www.nhc.gov.cn/yzygj/s7659/202002/dcf3333b740f4fabad5f9f908d1fc5b4.shtml)	Tools and approaches of functional evaluation based on ICF	Functional diagnosis and description based on ICF	Rehabilitation intervention based on ICHI
U07.100x001: Novel coronavirus pneumonia U07.100x002: Novel coronavirus infection U07.100x003: Suspected case of novel coronavirus pneumonia Z03.800x001: Suspected novel coronavirus pneumonia	WHO Disability Assessment Schedule 2.0 (36‐item version) Brief Model Disability Survey VB40 Generic Functions Domains Assessment of various physical functions, including cardiopulmonary function, limb function, cognitive function, psychological function, etc ADL evaluation Quality of life assessment	Body functioning (code B), the main body functions involved Body structure (code S), the main body structure involved Activity and participation (code D), main activities and participation involved Environment factor (code EF), environmental factors involved Personal factor (code PF), personal factors involved Healthy lifestyle and behavior	Preventive: Including physical activity, education, health consultation, etc Therapeutic: Including a variety of rehabilitation treatments, such as body position adjustment, expectoration treatment, physical therapy, respiratory training, psychological therapy, health‐care activities, sports therapy, ADL training, respiratory exercises, and other supportive treatments Health promotion: Including *taijiquan*, *wuqinxi*, *baduanjin*, and other physical activities, education, health consultation, health behavior consultation and services, etc

ADL, activities of daily living; ICD, International Classification of Diseases; ICF, International Classification of Functioning, Disability and Health; ICHI, International Classification of Health Interventions; WHO, World Health Organization; WHO‐FICs, World Health Organization Family of International Classifications.

### 2.2 Protocol of diagnosis, coding, evaluation, description and intervention of functioning of COVID‐19 cases based on ICF

[Consensus 2] The WHO recommends three standardized functional assessment tools based on the ICF in ICD‐11, namely the WHO Disability Assessment Schedule 2.0 (WHODAS 2.0), the Brief Model Disability Survey, and the Generic functioning domains (VB40). The functional evaluation based on the ICF, such as ICF‐Core set, can be used for patients’ overall functional assessment, rehabilitation‐needs assessment, and rehabilitation‐outcome assessment. This consensus recommends the use of these three standardized assessment tools in rehabilitation evaluation. The qualifiers of ICF can be used to standardize the results of functional assessment in the field of rehabilitation to comparable achieve comparable international functional data.

According to the ICF, this consensus also recommends all evaluations mapped to ICF structure involve in four aspects: body function and structure, activity and participation, environmental factors and personal factors.^15^, ^16^, ^17^, ^18^, ^19^, ^20^

According to the framework and scope of rehabilitation developed by the International Society of Physical and Rehabilitation Medicine (ISPRM white paper), ICF and ICHI β‐2, we develop a personalized intervention plan based on specific unmet needs of patients with COVID‐19.^21^, ^22^, ^23^, ^24^, ^25^, ^26^, ^27^

[Consensus 3] According to the menisfestion of COVID‐19 and related functioning, experts mainly recommend rehabilitation interventions related to lung function, physical activity, and psychological functions, which are widely used in chronic obstructive pulmonary disease (COPD). There were many recommendations in the rehabilitation clinical practice guidelines, and many studies provided relevant clinical evidences. The consensus also considers the environments in which interventions are applied, such as medical institutions, rehabilitation institutions, and communities, as well as the rehabilitation stage of patients. The clinical manifestation report from patients and the autopsy report for COVID‐19 are also provided evidences for this consensus.

Psychological intervention is an important part of comprehensive rehabilitation, which is of great significance for people with major diseases or functioning. This consensus also refers to the rehabilitation recomendations of psychological disorders caused by major disability, such as post‐traumatic stress disorder and spinal cord injury, as well as the WHO’s relevant guidelines and recommendations on natural disasters and COVID‐19, and develops psychological rehabilitation recommendations for patients with COVID‐19.

#### 2.2.1 Build COVID‐19 rehabilitation service delivery system based on ICF and WHO guideline: rehabilitation in health system.^6^, ^28^, ^29^, ^30^, ^31^

In light of the WHO rehabilitation guideine: Rehabilitation in health system, rehabilitation for COVID‐19 survivors should be provided at tertiary‐, secondary‐, and primary‐care levels and integrated into the continuum of prevention, treatment, rehabilitation, and health promotion.

[Consensus 4] According to the recommendations from WHO guidelines Rehabilitation in health system, considering the functioning caused by COVID‐19, including mental health issues and environmental support factors, we should implement the people‐oriented rehabilitation throughout lifespan and concerns social determinants, adopt multidisciplinary and cross‐domain approaches, and with approaches of universal design to bulid barriers‐free environments and to establish a comprehensive rehabilitation service system.

## 3 DIAGNOSIS AND CODING FOR COVID‐19 CASES^32^, ^33^, ^34^, ^35^, ^36^, ^37^, ^38^, ^39^

### 3.1 Virological characteristics

The new coronavirus is a coronavirus of β genus, with a capsule, round or oval particles, often pleomorphic, with a diameter of 60‐140 nm.^32^, ^33^, ^34^, ^40^ The virus is sensitive to UV; and heat of 56°C for 30 minutes, ether, 75% ethanol, chlorine containing disinfectant, peracetic acid, and chloroform can effectively inactivate the virus. Chlorhexidine cannot effectively inactivate the virus.

The novel coronavirus is the main source of infection. Asymptomatic infections can also be a source of infection. The main route of transmission is through respiratory droplets and close contact. It is possible to propagate through aerosols when exposed to high‐concentration aerosols for a long time in a relatively closed environment. In addition, the existing data cannot exclude the possibility of fecal oral transmission. People are generally susceptible to the virus. Based on the current epidemiological survey, the incubation period is 1‐14 days, mostly 3‐7 days, and the longest period may be 24 days.^1^, ^19^

### 3.2 Pathological characteristics

The results of a lung biopsy and autopsy in a COVID‐19 patient showed that the patient had pleural effusion, pleural thickening, and extensive and severe adhesion with the lung. The lung tissue showed dark red and gray white patchy changes in toughness, while a large amount of gray white viscous fluid overflowed in the lung tissue, and fiber cords were seen. White foam mucus was seen in the endotracheal tube, and mucus adhered in the lumen of the right pulmonary branch.

### 3.3 Clinical manifestations

The main manifestations of patients are fever, dry cough, and fatigue. A small number of patients have nasal obstruction, runny nose, sore throat, myalgia, diarrhea, and other symptoms. Severe patients often have dyspnea and/or hypoxemia 1 week after the onset of the disease. Severe patients can rapidly progress to acute respiratory distress syndrome, septic shock, metabolic acidosis, coagulation dysfunction, and multiple organ failure. It should be noted that the course of severe and critical patients may be moderate to low fever, or even no obvious fever. Mild patients only show low fever, slight asthenia, and no pneumonia.

It is necessary to establish a multi‐disciplinary rehabilitation team for patients with COVID‐19. According to the functioning characteristics and the rehabilitation stage of patients, individualized rehabilitation intervention strategies and approached are recommended.^5^, ^41^ From the current situation of the cases, most patients have a good prognosis, and a few patients are in a critical condition. The prognosis of the elderly and those with chronic basic diseases is poor. The symptoms of children are relatively mild.

### 3.4 Clinical classification

Mild: The clinical symptoms are mild, and no pneumonia is found in radiology.

Moderate: Fever, respiratory tract and other symptoms are present, and pneumonia is found in radiology.

Severity: Those complying with any of the following should be considered as having the heavy type: (1) when shortness of breath occurs, the respiratory rate is ≥30/min; (2) under the resting state, the oxygen saturation is ≤93%; (3) arterial oxygen partial pressure (PaO_2_)/fraction of inspiration O_2_ (FiO_2_) is <300 mm Hg (1 mm Hg = 0.133 kPa); (4) radiology shows that the lesions progress more than 50% in 24‐48 hours.

Extreme Severity and critical statues: Those meeting one of the following conditions should be treated as severe: (1) respiratory failure occurs and requires mechanical ventilation; (2) shock; and (3) other organ failure occurs and requires intervention in intensive care unit.

### 3.5 Diagnosis and coding of COVID‐19 with ICD‐11 and ICD‐10

#### 3.5.1 Coding protocol of COVID‐19 with ICD‐11 and ICD‐10 recommended by WHO 

WHO recommended to use U07.1 code for virus idendtified COVID‐19, and to use U07.2 code for virus not identified, including clinically‐epidemiologically diagnosed COVID‐19, probable COVID‐19, and suspected COVID‐19 cases in ICD‐10. In ICD‐11, the code for the confirmed diagnosis of COVID‐19 is RA01.0 and the code for the clinical diagnosis (suspected or probable) of COVID‐19 is RA01.1 (https://www.who.int/classifications/icd/covid19/en/).

#### 3.5.2 Coding protocol of COVID‐19 with ICD‐10 recommended by China National Health and Health Commission and China National Health Insurance Bureau 

A coding protocol of COVID‐19 with ICD‐10 had been issued by National Health and Health Commission and National Health Insurance Bureau.

The “U07.100” code is used for novel coronavirus as a statistical code for all confirmed COVID‐19 cases. It is not used as a main diagnostic code for the front page of the medical record.

##### Code U07.100x001: Novel coronavirus pneumonia

The “U07.100x001: Novel coronavirus pneumonia” code is only applicable to the “confirmed inpatients with new coronavirus pneumonia” and must be used as the main diagnostic encode.

The code is defined as follows: (1) It can only be used for confirmed cases of COVID‐19. For suspected cases, one of the following examples of etiological evidence is required: positive real‐time fluorescence RT‐PCR detection of new coronavirus nucleic acid; and/or virus gene sequencing highly homologous with the new coronavirus. Note: The novel coronavirus infection diagnosis is not limited to respiratory tract specimens or blood samples. Urine, stool, and conjunctiva specimens can be used as diagnostic evidence. The specific diagnostic criteria are detailed in the Novel coronavirus pneumonia treatment and treatment plan (trial version 7). (2) It can only be used for a confirmed COVID‐19 case in hospital, excluding outpatient, observation, shelter, home quarantine cases, etc.

##### Code U07.100x002: Novel coronavirus infection

The “U07.100x002: Novel coronavirus infection” code is only applicable to “confirmed inpatients (excluding new coronavirus pneumonia)” and is used as the main diagnostic code.

Code definition: (1) The novel coronavirus nucleic acid test is positive, but did not induce pneumonia in “atypical” patients. For example, no novel coronavirus pneumonia‐related clinical manifestations, or symptoms of gastrointestinal symptoms (such as diarrhea, nausea, vomiting), heart‐related symptoms (such as palpitations), or comprehensive neurological symptoms (such as headache) are present. (2) The case had been diagnosed with the novel coronavirus pneumonia and is in hospital, excluding outpatient, observation, shelter, and home quarantine cases.

##### Code U07.100x003: Suspected case of novel coronavirus pneumonia

The “U07.100x003: Suspected case of novel coronavirus pneumonia” code is only applicable to suspected cases of new coronavirus pneumonia in hospital.

Code definition: (1) The case is a new coronavirus or nucleic acid or gene test negative, and conforms to any one of the following epidemiological histories or any two clinical manifestations, or no clear epidemiological history, accompanied by any of three clinical manifestations. (2) The case is a suspect case with novel coronavirus pneumonia for hospitalization, excluding outpatient service, observation, shelter, home quarantine cases, and so on.

##### Code Z03.800x001: Observed case of novel coronavirus pneumonia

The “Z03.800x001: Observed case of novel coronavirus pneumonia” code is only applicable to patients who have been discharged from hospital, but had been inpatients because of the new coronavirus pneumonia symptoms or signs before. The code should only be used as other diagnostic encode.

Code definition: (1) The patient must be considered as having novel coronavirus pneumonia, and accompanied by any one of the following epidemiological histories, or no significant epidemiological history, or any 1‐2 of the clinical manifestations. (2) The patient must have been excluded from having novel coronavirus pneumonia during the hospital stay, for example, two or more nucleic acid tests are negative. (3) The patient must be in hospital. They do not include outpatient service, observation, shelter, home quarantine cases, and so on.

### 3.6 Description and coding of functioning of cases with COVID‐19

[Consensus 5] COVID‐19 cases have secondary functioning and disability. Using the rapid extended ICF core set, we can describe, evaluate, and code the functioning of cases with COVID‐19. This rapid ICF core set developed from ICF core set for COPD. See Table 2 for description and coding demonstration case.

**TABLE 2:** Coding and description of functioning for cases with COVID‐19

	Definition and code	Function description
body function	Consciousness functions (b110)	General mental functions of the state of awareness and alertness, including the clarity and continuity of the wakeful state
	Energy and drive functions (b130)	General mental functions of physiological and psychological mechanisms that cause the individual to move towards satisfy specific needs and general goals in a persistent manner
	Sensation of pain (b280)	Sensation of unpleasant feeling indicating potential or actual damage to some body structure
	Immunological system functions (b435)	Functions of the body related to protection against foreign substances, including infections, by specific and non‐specific immune responses
	Respiratory functions (b440)	The function of breathing air into the lungs, exchanging air with blood, and exhaling air
	Respiratory muscle functions (b445)	The function of muscles involved in breathing
	Additional functions of the respiratory system (b450)	Additional functions related to breathing, such as producing and transporting airway secretions, coughing, sneezing and yawning
	Sensations associated with cardiovascular and respiratory functions (b460)	Sensations such as missing a heart beat, palpitation and shortness of breath
	Ingestion functions (b510)	Functions related to taking in and manipulating solids or liquids through the mouth into the body
Body structure	Lungs (S4301)	
	Structure of trunks (S760)	
Activities and participation	Speaking (d330)	Producing words, phrases and longer passages in spoken messages with literal and implied meaning, such as expressing a fact or telling a story in oral language
	Changing basic body position (d410)	Getting into and out of a body position and moving from one location to another, such as getting up out of a chair to lie down on a bed, and getting into and out of positions of sitting, standing, kneeling or squatting
	Transferring oneself (d420)	Moving from one surface to another, such as sliding along a bench or moving from a bed to a chair, without changing body position
	Caring for body parts (d520)	Looking after those parts of the body, such as skin, face, teeth, scalp, nails and genitals, that require more than washing and drying
	Dressing (d540)	Carrying out the coordinated actions and tasks of putting on and taking off clothes and footwear in sequence and in keeping with climatic and social conditions, such as by putting on, adjusting and removing shirts, skirts, blouses, pants, undergarments, saris, kimono, tights, hats, gloves, coats, shoes, boots, sandals and slippers
Environmental factor	Products or substances for personal consumption (e110)	Any natural or human‐made object or substance gathered, processed or manufactured for ingestion
	Products and technology for personal use in daily living (e115)	Equipment, products and technologies used by people in daily activities, including those adapted or specially designed, located in, on or near the person using them
	Products and technology for personal indoor and outdoor mobility and transportation (e120)	Equipment, products and technologies used by people in activities of moving inside and outside buildings, including those adapted or specially designed, located in, on or near the person using them
	Immediate family (e310)	Individuals related to birth, marriage, or other cultural traditions that are recognized as members of the immediate family, such as spouses, parents, siblings, children, foster parents, stepparents, and grandparents
	Social security services, systems and policies (e570)	Services, systems and policies aimed at providing income support to people who, because of age, poverty, unemployment, health condition or disability, require public assistance that is funded either by general tax revenues or contributory schemes
	Health services, systems and policies (e580)	Services, systems and policies for preventing and treating health problems, providing medical rehabilitation and promoting a healthy lifestyle

## 4 THE PRINCIPLES OF FUNCTIONAL EVALUATION AND DESCRIPTION

[Consensus 6] According to the disease classification, functional status, and rehabilitation needs of patients with COVID‐19, the following rehabilitation assessment and evaluation are recommended.

As novel coronavirus is highly infectious and highly pathogenic, any rehabilitation assessment and intervention must first ensure the safety of rehabilitation personnels and the reasonable use of protective equipment. Use of systematic and comprehensive functional assessment and evaluations is recommended. When equipment assessment is necessary, effective protective measures should be ensured. Elderly, severe, extreme severe and critical patients may be complicated with multiple basic diseases or direct invasion of multiple organs and systems. If necessary or after discharge, comprehensive assessment and evaluation are recommended to develop a rehabilitation plan to improve intervention and outcome.^23^

### 4.1 Comprehensive evaluation of functioning using ICF‐based tools

We recommend the use of standardized functional assessment tools based on the ICF to control quality of rehabilitation medical treatment, standardize the reporting of outcome, conduct functioning‐related statistical analysis, and improve the comparability of evidence‐based data. There are many functional assessment tools in the fields of rehabilitation. The ICD‐11 recommends three assessment tools in the supplementary chapters: the WHODAS 2.0,^35^, ^42^ the Brief Model Disability Survey,^36^, ^42^ and the VB40 general functional areas. We recommend WHODAS 2.0 for comprehensive evaluation of functioning for COVID‐19 cases during rehabilitation. WHODAS 2.0 36‐item version has six dimensions: Cognition, Self‐care, Getting along, Life Activities, and Participation. It can be used: (1) as a tool for evaluating the overall function of patients in the rehabilitation of novel coronavirus pneumonia; (2) on the front page of the medical record to report the functional outcome of patients; and (3) for statistcis of rehabilitation outcome.

The ICF core set is a widely used standardized tool. There is no ICF core set for COVID‐19. We recommend a rapid and extended ICF core set from ICF core set for COPD for evaluation and description of functioning and disability for COVID‐19 cases (Table 2).

### 4.2 Assessments and evaluations in body structure and function

According to the COVID‐19‐cases‐related body functions and structures and the assessments and evaluation tools commonly used in the fields rehabilitation, we recommend the following assessments and evaluations.

#### 4.2.1 Recommended subjective assessments and evaluations in body structure and function

(1) Evaluation of dyspnea: Modified Borg Scale for daily follow‐up.
(2) Subjective fatigue assessment: Rating of Perceived Exertion.
(3) Limb pain assessment: Visual Analog Scale and Oral Rating Scales.
(4) Evaluation of anxiety and depression: Zung’s Anxiety/Depression Scales, Self‐rating Depression Scale and Self‐rating Anxiety Scale for regular follow‐up evaluation.

#### 4.2.2 Recommended clinical examinations assessments and evaluations in body structure and function

(1) Vital signs evaluation: Record the body temperature, respiration, pulse, blood pressure, blood oxygen saturation, urine volume, and other indicators regularly every day, and make relevant records before and after the intervention treatment.
(2) Imaging evaluation: Chest X‐ray; if necessary, CT and color Doppler echocardiography may be used to evaluate the morphology of heart and lung and provide an objective basis for making a treatment plan.
(3) Assessment of bone, joint and muscle: Bed rest or disease consumption would reduce intake, and other reasons may lead to the decreased function of skeletal muscle, soft tissue level, and joint stiffness. It can regularly monitor the limb muscle circumference, subcutaneous fat thickness, grip strength, active or passive range of motion. As a simple index, grip strength can be closely related to prognosis and can be widely used in patients.
(4) Motor function evaluation: According to the stability of the patient’s condition, carry out balance function test in the early stage or when necessary, such as one‐leg closed‐eyes time, 10‐m walking test, 6‐minute walking test, daily walking speed test, and other comprehensive evaluation tests of cardiopulmonary function should be implicated when patients are in the recovery period, discharged, or undergoing follow‐up outside the hospital. A 6‐minute walking test or cardiopulmonary exercise test can also be implicated, if necessary. Strength, Assistance with walking, Rise from a chair, Climb stairs and Falls can provide more information, such as balance, coordination ability, and so on, but requires a higher functional level of patients.
(5) Pulmonary function evaluation: Patients followed up outside the hospital (especially those who experienced pulmonary exudation during the disease) should be assessed for their lung‐function test, which is helpful to understand the recovery of ventilation function. In cases where patients may still have the ability to transmit the virus, the examination should be avoided. If necessary, an individual handheld pulmonary function instrument may be used. In addition, dynamic lung capacity can also be used for evaluation of the expiratory flow rate per unit time, which can better reflect airway resistance.

Diaphragm function assessment: COVID‐19 may not directly invade the diaphragm. However, some patients may have insufficient diaphragm function and diaphragm fatigue due to factors such as increased ventilation demand, decreased efficiency, bed rest, consumption of disease, and intake reduction. Therefore, inspiratory peak flow rate, and ultrasound diaphragm activity and thickness can be used for diagnosis according to patient’s stability and treatment needs. Direct or indirect evaluation can provide more reference information for patients with decreased activity tolerance and difficulty in offline.

### 4.3 Evaluations in activity and participation

We recommend the WHODAS 2.0, activities of daily living (ADL) assessments, and the 36‐item Short Form Health Survey (SF‐36) to evaluate patients’ activities of daily life and participation.

#### 4.3.1 WHODAS 2.0

The WHODAS 2.0 is a standardized activity and participation assessment tool recommended by the WHO.

#### 4.3.2 ADL

According to the severity of the patient’s condition, ADLs should be evaluated regularly for mild, medium, severe, and discharged patients, including basic ADL evaluation and instrumental ADL evaluation for cases who return to the community after discharge. The improved Barthel Index or other instrumental ADL scales are recommended. For severe cases, we recommend observational evaluation.

#### 4.3.3 Quality of life

As there are no assessment scales specific to COVID‐19, we recommend using the Medical Outcomes Study SF‐36 or the COPD Assessment Test in order to shorten the time of evaluation.

### 4.4 Assessments and evaluations at different stages of rehabilitation

Considering that COVID‐19 cases may have different health conditions and will be at different rehabilitation stages, we recommend not only collecting data of vital signs, laboratory tests, and other information on disease, but also evaluating pulmonary function, subjective fatigue, dyspnea, pain, joint and muscle function, activity level, and quality of life tailored to patients’ health conditions. Those assessments should be carried out pre‐ and post‐rehabilitation. Only comprehensive and systematic rehabilitation data will provide evidence for the effect of rehabilitation in the intervention of infectious diseases, such as COVID‐19.

## 5 PROTOCOL OF COVID‐19 REHABILITATION INTERVENTION BASED ON ICF

[Consensus 7] We developed a protocol of rehabilitation intervention based on the ICF tailored to COVID‐19 diagnosis, functional status, and unmet needs of rehabilitation.

### 5.1 Rehabilitation environment and setting, measures, and principles

Within the framework of the WHO‐FICs, rehabilitation service delivery for COVID‐19 cases can be implemented in hospitals, rehabilitation institutions, and communities (such as primary‐care and community health‐service institutions). Rehabilitation measures may include: preventive, therapeutic, health‐promoting, and palliative care according to their intervention purposes.^5^ It was proposed to develop individualized rehabilitation plan for COVID‐19 cases in light of patients’ specific health conditions, functional characteristics, and unmet needs of rehabilitation in different stages of rehabilitation. Therapeutic methods are mainly used in hospitals (inpatients). Preventive and health‐promotion methods can be provided for patients after discharge and as outpatient care as well as for those in the community. Rehabilitation doctors and therapists should develop individualized rehabilitation programs according to the actual health condition and functional state, the expected outcomes, the expectations of patients and their families, and the actual services setting.

### 5.2 Pulmonary function rehabilitation

#### 5.2.1 Intensive rehabilitation training

The main clinical manifestations of COVID‐19 are respiratory dysfunction, with pulmonary consolidation and airway secretion obstruction.^43^ As part of non‐drug therapy, lung rehabilitation can play an important role in the acute stage. Novel coronavirus pneumonia is a new disease. In the absence of evidence for clinical treatment and rehabilitation of the disease, when making treatment targets and measures, we should consider the pathological changes of pleural effusion, pleural adhesion, increased airway secretion, and obstructive atelectasis in COVID‐19. We suggest that thorax mobilizing therapy, expectoration therapy, respiratory training, and so forth should be applied. Rehabilitation interventions should improve patients’ symptoms and clinical outcomes.

#### 5.2.2 Time selection of rehabilitation intervention

At present, there is no clinical evidence for the optimal time of rehabilitation intervention for the disease. The benefits and risks of patients should be balanced and cross‐infection should be avoided. For patients with severe or critical illness, rehabilitation treatment should be carried out when vital signs are stable, and the changes of vital signs should be monitored throughout the process. Rehabilitation doctors and therapists should strengthen interdisciplinary communication, and devise and implement intervention treatment together with clinicians. During the operation, therapists can use the early warning scores or the Awakening and Breathing Coordination, Delirium Monitoring/Management, and Early Exercise/Mobility Bundle (ABCDE)^44^ Scale to evaluate the safety of rehabilitation treatment in real time and avoid increasing iatrogenic damage.

### 5.3 Expectoration coach

According to the patient’s condition, the therapist can use body‐position drainage, vibration and clapping, active cycle of breathing techniques, and other techniques or equipment to clear the airway, and should pay attention to the local humidification of the whole body and airway to reduce the viscosity of the sputum.

### 5.4 Position adjustment

For patients with dyspnea, cough, and other symptoms, an upright sitting or standing position can increase diaphragm activity, improve the Ventilation/Perfusion in the lung, increase tidal volume, improve peak flow rate of cough, and reduce the sense of breathing difficulty. When the secretion of the respiratory tract increases, the therapist can adjust the posture according to the change of vital signs, and assist in expectoration. In the intensive care unit, an electric vertical roll bed is recommended to help patients adjust their position.

For mild, medium, and heavy patients, and patients after discharge, reducing the time spent in bed is helpful to reduce the risk of various bed complications, promote the improvement of cardiopulmonary function, and shorten recovery time.

### 5.5 Breathing training

The threshold loading inspiratory muscle training device is the most commonly used method of respiratory resistance training at present. Generally, the initial load is 30% of the personal maximum inspiratory pressure. According to the training purpose (endurance training or strength training), a gradual increase in the training load or training times is recommended, along with training 1‐2 times a day, 15‐30 rounds each time (for endurance training), or 8‐12 rounds each time (for strength training).^27^

Respiratory control can significantly improve the experience of breathing, reduce respiratory‐related oxygen consumption, and relieve the tension of patients. Generally, deep and slow breathing is used to increase the compliance of the respiratory system, reduce the work of breathing, and relieve the dyspnea of patients. Diaphragmatic breathing or abdominal breathing also have similar effects. If possible, physical therapy can be used for training, such as an electrical stimulation diaphragmatic trainer, electronic biofeedback, and so forth.

### 5.6 Exercise therapy

Exercise training is the basis of various rehabilitation measures. It can not only effectively improve the functional level of the cardiopulmonary muscle unit, reverse the decline of disuse function, but also increase the compensatory ability of the noninvolved organs, and provide more functional reserves for the disease. At present, it mainly includes aerobic (endurance) training, resistance (strength) training, balance training, and coordination training. According to the evaluation results, the four elements (frequency, intensity, time, and type) of exercise prescription and the three principles (reasonable overload, repeatability, and specificity) should be followed in the formulation of the plan, and individualized and precise prescription should be formulated to avoid sports injury while pursuing benefits.^43^, ^44^, ^45^, ^46^, ^47^, ^48^

#### 5.6.1 Aerobic (endurance) training

In aerobic training, it is best to monitor blood pressure, heart rate, and blood oxygen saturation.

(1) Intensity: Patients in the acute stage mainly take low‐intensity exercise without fatigue on the second day. In general, patients in better condition can try medium‐intensity exercise. After discharge, patients can carry out medium‐ and high‐intensity exercise training according to further evaluation results to obtain more benefits.
(2) Frequency: According to the patient’s tolerance, they should carry out active and passive training once or twice a day. If the patient’s condition is serious and the tolerance is poor, they should shorten the training duration and increase the frequency accordingly to ensure the training quantity.
(3) Time: Patients with good state and those discharged from hospital can accumulate aerobic exercise time to 60 minutes every day, and those in good condition can increase the time moderately; for patients with heavy or critical disease, exercise should be accumulated to about 30 minutes every day, if possible.
(4) Activity mode: A patient should carry out fast walking, slow walking, standing, sitting upright, and other sports modes according to the environmental conditions and their own condition.

#### 5.6.2 Resistance movement

(1) Intensity: Patients in the acute stage should take low‐ and medium‐ intensity load training with the goal of maintaining muscle mass or delaying muscle atrophy. If possible, they can start from 30% to 40% of 1 RM, and if not, they can use 1‐5 kg. According to the patient’s tolerance degree and the change of the condition, the load or bodyweight training should be gradually increased; after discharge, the patients may use medium‐ and high‐intensity load training with the goal of recovering or increasing muscle mass. The effective prescription for improving muscle strength was 65% 1 RM for three groups, three times a week. Load training for patients with novel coronavirus pneumonia should be reduced as appropriate.
(2) Frequency: Low‐ and medium‐intensity load trainers can try 1‐2 times a day, and medium‐ and high‐intensity load trainers can try one time every other day.
(3) Methods: Progressive resistance training is a commonly used program. Resistance load can come from self‐weight, external resistance (such as elastic band, sandbag, dumbbell, etc). There are many application videos of elastic band resistance training on the internet, which can be used to carry out alternating and repeated training of large muscle groups of limbs.

#### 5.6.3 Joint active and passive motion

Long‐term bed rest can lead to joint stiffness, contracture, and other changes. Patients should be guided to actively carry out active and passive motions of the spine and limb joints to maintain their normal range of motion, which can be arranged 1‐2 times per day and can be completed by joint and position. Severe patients may not be able to complete the active whole joint motion effectively. At this time, they may need other people or special equipment to carry out the passive motion of joints, including the limbs, head, neck, and waist, to reduce the risk of deep vein thrombosis.

### 5.7 Physical factor therapy

(1) Ultrashort wave therapy: Ultrashort wave therapy can promote the exudation and absorption of the lung and improve ventilation function. It can be used for patients with lung exudation and the specific prescription is mainly short‐term with micro or without heat; however, fever is a contraindication. Small ultrashort waves can be used for bedside treatment to reduce the impact of high‐frequency electromagnetic fields on monitoring equipment, but the ultrashort wave machine, its electrodes, and wires should be disinfected and protected according to the protection level.
(2) Ultraviolet therapy: Whole‐body ultraviolet irradiation can increase immunity function, which may be applicable to mild and medium patients, but severe and critical patients may have immune disorders, so it is not recommended.
(3) Low‐frequency neuromuscular electrical stimulation therapy: Neuromuscular electrical stimulation can improve the respiratory muscle and peripheral muscle function, so it can be used in bed patients for respiratory muscles or limb muscles, to delay muscle disuse atrophy, and to increase neuromuscular function.

### 5.8 Deep‐breathing exercises

Deep‐breathing control is a special form of training. It can be skillfully mastered by patients in a short period of time through a series of carefully choreographed action routines (taking into account a variety of treatment purposes, such as emotional regulation, low‐intensity aerobic training, and respiratory muscle group resistance training) and with the help of words, pictures, videos, and other communication methods, which is very suitable for patients. During breathing training, it is necessary to pay attention to the coordination of diaphragm movement with trunk and limb movement so that diaphragm‐function training, breathing‐mode training, and body and joint training can be carried out at the same time.

### 5.9 Psychological intervention

For some COVID‐19 cases, there may be some negative stress responses, mainly emotional disorders, such as panic, anxiety, and somatization symptoms, which affect the mood, state, sleep and overall mental health level.^49^, ^50^ These psychological and behavioral disorders will affect the treatment effect of patients.

For hospitalized patients with emotional disorders, such as anxiety or depression, we recommend: the implementation of psychological intervention as early as possible (including cognitive therapy and behavior therapy); the elimination of stressors; the improvement of patients’ anxiety or depression; establishing a positive and optimistic mood; and seeking support from families, medical staff, and psychologists. In addition to psychological and behavioral interventions, drug therapy and biofeedback therapy can also be used in the treatment of severe anxiety or depression.

For COVID‐19 patients who receive rehabilitation in the community, we recommend: the establishment of a psychological support service system; the relief of panic about infectious diseases at the community level; the establishment of a psychological support and assistance system between community members, family members and patients; and the provision of special psychological services for those who suffer critical psychological events in the pandemic situation, such as the death of family members, to ensure that patients will not suffer from serious psychological obstacles that reduce their quality of life.

In view of the psychological problems caused by COVID‐19, we recommend to provide mental health services for patients and their families, assist them to acquire and understand the correct information about the pandemic and the impact of COVID‐19, prevent them from panic and long‐term stress state, and help them to establish a positive lifestyle and behaviors.

### 5.10 Health‐promotion activities

Participating in *taijiquan*, *wuqinxi*, *baduanjin*, and other physical activities is helpful to regulate breath, dredge meridians, and improve stability of the core muscle group and balance ability. These activities are especially suitable for elderly or weak patients with low physical abilities and can be carried out by mild, medium, and discharged patients, using group mutual aid mode or at home through video exercises.

### 5.11 Interruption criteria of rehabilitation intervention

We recommend the following criteria for interruption of intervention for COVID‐19 cases with slight and medium functioning if one of the following conditions occurs: (1) there is obvious fatigue or a Borg Dyspnea Score >3, which cannot be relieved after rest; or (2) chest tightness, suffocation, dizziness, headache, unclear vision, palpitation, sweating, inability to maintain balance, and so forth.

For COVID‐19 cases with one of the following conditions, we recommend the immediate cessation of intervention: (1) Respiratory system: blood oxygen saturation ≤90% or more than 4% lower than the baseline value; respiratory frequency >40 times/min; dyspnea or shortness of breath, aggravation, fatigue, and intolerable fatigue. (2) Cardiovascular system: systolic pressure <90 mm Hg or >180 mm Hg; mean arterial pressure <65 mm Hg or >110 mm Hg, or more than 20% change from baseline; heart rate <40 times/min or >120 times/min; new arrhythmia and myocardial ischemia. (3) Nervous system: poor state of consciousness.

## 6 REHABILITATION MANAGEMENT AND GOVERNANCE

[Consensus 8] Considering the importance of rehabilitation management and governance in modern rehabilitation services, we have developed a protocol of rehabilitation management and governance using the WHO‐FICs framework for COVID‐19 cases considering the functioning characteristics. This model is multidisciplinary and across sectors, covering health continuum of prevention, treatment, rehabilitation, and health promotion. COVID‐19 cases can receive rehabilitation services in hospitals, rehabilitation institutions, and community primary care institutions. These patients need to receive life span rehabilitation to improve their overall functioning and their quality of life. Community rehabilitation service is the main extended form of institutional rehabilitation service. Rehabilitation management is an important mechanism to ensure that patients receive high‐quality rehabilitation services from medical institutions, rehabilitation institutions, and community based service providers. We should build a patient‐centered rehabilitation service system.

It is necessary to establish a multidisciplinary rehabilitation team for patients with COVID‐19. According to the functioning characteristics and the rehabilitation stage of patients, individualized rehabilitation intervention strategies and approaches are recommended.^5^, ^41^

We should establish a comprehensive rehabilitation service system in all professional fields, integrating all levels of health services, and providing comprehensive rehabilitation services for COVID‐19 patients from clinical treatment to community rehabilitation services. In the community, we should especially emphasize the empowerment of patients, enhance their self‐confidence, and improve their overall functions and their quality of life.

### 6.1 Establish a comprehensive rehabilitation system 

For patients in different rehabilitation stages, we should analyze their unmet needs of rehabilitation, main functioning disability and rehabilitation resources from medical institutions, rehabilitation institutions and communities levels, and establish different rehabilitation solutions to improve the quality and cost‐effectiveness of rehabilitation.^5^

### 6.2 Using big data and remote rehabilitation and other new technology

We recommend: the establishment of a rehabilitation service platform; cooperation with experts in epidemiology and clinical medicine; and the integration of rehabilitation into the modern health‐service system. For special patient groups, such as the elderly, people with disabilities, and children, we strongly recommend the consideration of their special needs and obstacles, as well as multiple functioning and its impact on COVID‐19 rehabilitation.

In the community, we recommend the all members pay attrention to the negative influences and discrimination against COVID‐19 cases. The proposed measures include the provision of correct information, the prevention of panic and bias, and the consideration of the psychological, social, and environmental factors in community rehabilitation services.^41^

## CONFLICTS OF INTEREST

Nothing to disclose.

## Appendix 1

### Members of the expert committee

Senior Advisors: Professor Jianye Wang (Beijing Hospital, National Center of Gerontology), Pulin Yu (Beijing Hospital, National Center of Gerontology), Xianguang Wu (Research Institute of Rehabilitation Information, China Rehabilitation Research Institute/China Rehabilitation Research Center; Division of Disability Classification Research of China Association of Rehabilitation of Disabled Persons).

Members: Bingchen An (East China Hospital Affiliated to Fudan University), Dingqun Bai (The First Hospital Affiliated to Chongqing Medical University), Di Chen (Research Institute of Rehabilitation Information, China Rehabilitation Research Institute/China Rehabilitation Research Center; World Health Organization Collaborating Center of Family International Classifications in China; Division of Disability Classification Research of China Association of Rehabilitation of Disabled Persons), Zhuoming Chen (The First Affiliated Hospital of Jinan University), Jingyuan Deng (The First Affiliated Hospital of Xi’an Jiaotong University), Qi Guo (Shanghai University of Medicine & Health Sciences), Chengqi He (Huaxi Hospital of Sichuan University), Xiquan Hu (The Third Affiliated Hospital of Zhongshan University), Chongxia Huang (Guangzhou Zhenggu Hospital), Qiuchen Huang (Research Institute of Rehabilitation Information, China Rehabilitation Research Institute/China Rehabilitation Research Center), Xuming Huang (The First Affiliated Hospital of Guangdong Pharmaceutical University), Zhen Huang (Panyu Central Hospital), Xinping Li (Guangdong People’s Hospital/Guangdong Academy of Medical Sciences), Zhongming Liang (The Third People’s Hospital of Guiyang), Gang Liu (The Third Affiliated Hospital of Southern Medical University), Peng Liu (The First Affiliated Hospital of Zhongshan University), Chao Ma (Sun Yat‐Sen Memorial Hospital of Sun Yat‐sen University), Hongzhuo Ma (World Health Organization Collaborating Center of Family International Classifications in China; China Academy of ICF of Weifang Medical University; Division of Rehabilitation Psychology of Chinese Psychological Society; Division of Disability Classification Research of China Association of Rehabilitation of Disabled Persons), Zhongxiang Mi (China Rehabilitation Research Center/Medical Rehabilitation Institution Management division of China Hospital Association), Cuihuan Pan (The Second Affiliated Hospital of Guangzhou Medical University), Zhuoying Qiu (Research Institute of Rehabilitation Information, China Rehabilitation Research Institute/China Rehabilitation Research Center; World Health Organization Collaborating Center of Family International Classifications in China; China Academy of ICF of Weifang Medical University; Division of Rehabilitation Psychology of Chinese Psychological Society; Division of Disability Classification Research of China Association of Rehabilitation of Disabled Persons), Xiue Shi (Shanxi Rehabilitation Hospital), Hongwei Sun (China Academy of ICF of Weifang Medical University; World Health Organization Collaborating Center of Family International Classifications in China; Division of Rehabilitation Psychology of Chinese Psychological Society; Division of Disability Classification Research of China Association of Rehabilitation of Disabled Persons), Guoxiang Wang (School of Physical Education/Institute of Exercise Rehabilitation and Sport Sciences of Soochow University; World Health Organization Collaborating Center of Family International Classifications in China; China Academy of ICF of Weifang Medical University; Division of Disability Classification Research of China Association of Rehabilitation of Disabled Persons), Jianing Xi (Beijing Rehabilitation Hospital Affiliated to Capital Medical University/Rehabilitation Organization Management Committee of Chinese Rehabilitation Medical Association), Xiaofei Xiao (Binzhou Medical University), Tao Xu (Rehabilitation Medicine Department, Tongji Hospital, Tongji Medical College, Huazhong University of Science and Technology), Wuhua Xu (Guangzhou Red Cross Hospital), Jian Yang (School of Physical Education and Health of East China Nomal University; World Health Organization Collaborating Center of Family International Classifications in China; China Academy of ICF of Weifang Medical University; division Division of Rehabilitation Psychology of Chinese Psychological Society; Division of Disability Classification Research of China Association of Rehabilitation of Disabled Persons), Shaohua Yang (Affiliated Hospital of Guilin Medical College), Wanzhang Yang (Shenzhen Hospital Affiliated to Southern Medical University), Xiangming Ye (Zhejiang People’s Hospital), Xiaoping Yun (Beijing Boai Hospital of China Rehabilitation Research Center), Bin Zeng (Guangdong People’s Hospital/Guangdong Academy of Medical Sciences), Aiming Zhang (Research Institute of Rehabilitation Information, China Rehabilitation Research Institute/China Rehabilitation Research Center; Division of Disability Classification Research of China Association of Rehabilitation of Disabled Persons), Chong Zhang (The First Affiliated Hospital of Guangxi University of Traditional Chinese Medicine), Minsheng Zhang (Guangdong People’s Hospital/Guangdong Academy of Medical Sciences), Pande Zhang (The First People’s Hospital of Foshan), Qiaojun Zhang (The Second Affiliated Hospital of Xi’an Jiaotong University), Mingming Zhao (Guangxi Jiangbin Hospital), Jiejiao zheng (East China Hospital Affiliated to Fudan University).

## References

[1] Office of China National Health and Health Committee, Office of the State Administration of Traditional Chinese Medicine .Novel coronavirus pneumonia treatment and treatment plan (trial version 7): NO.184. http://www.gov.cn/zhengce/zhengceku/2020‐03/04/5486705/files/ae61004f930d47598711a0d4cbf874a9.pdf. Published March 3, 2020. Accessed March 3, 2020.

[2] Yang X , Yu Y , Xu J , et al. Clinical course and outcomes of critically ill patients with SARS‐CoV‐2 pneumonia in Wuhan, China: a single‐centered, retrospective, observational study. Lancet Respir Med. 2020;8(5):475‐481. 32105632 10.1016/S2213-2600(20)30079-5 PMC7102538

[3] Guan W‐J , Ni Z‐Y , Hu Y , et al. Clinical characteristics of coronavirus disease 2019 in China. N Engl J Med. 2020;382(18):1708‐1720. 32109013 10.1056/NEJMoa2002032 PMC7092819

[4] World Health Organization . World Health Organization Family International Classifications, WHO‐FICs. https://www.who.int/classifications/en/. Accessed February 2, 2012.

[5] Qiu Z‐Y , Li L , Chen D . Research on rehabilitation guidelines using World Health Organization Family International Classifications: framework and approaches. Chin J Rehabil Theory Pract. 2020;26(2):125‐135.

[6] Qiu Z‐Y , Kwok J , Yang J . Rehabilitation 2030: realization of United Nation sustainable development goals 2030. Chin J Rehabil Theory Pract. 2017;23(4):373‐378.

[7] World Health Organization . Groups that were involved in ICD‐11 Revision Process. https://www.who.int/classifications/icd/revision/en/. Accessed March 20, 2020.

[8] World Health Organization . International Classification of Functioning, Disability and Health (International Chinese Supplement). Geneva, Switzerland: World Health Organization; 2015.

[9] Qiu Z‐Y , Chen D , Chen Y . Construction of knowledge system of modern sciences of rehabilitation and rehabilitation education regarding ICF Model. Chin J Rehabil Theory Pract. 2009;15(12):1193‐1195.

[10] Stucki G , Melvin J , Lu W , Li Z , Qiu Z‐Y . International Classification of Functioning, Disability and Health: a model for the unified conceptual description of physics and rehabilitation medicine. Chin J Rehabil Theory Pract. 2008;14(12):1108‐1111.

[11] Stucki G , Grimby G . Building a special subject area for human function and rehabilitation research: developing a comprehensive structure from cell to society. Chin J Rehabil Theory Pract. 2008;14(12):1112‐1115.

[12] Grimby G , Melvin J , Stucki G . The development and application of ICF: the construction of rehabilitation knowledge system and clinical tools. Chin J Rehabil Theory Pract. 2008;14(12):1101‐1102.

[13] World Health Organization . International Classification of Health Interventions (ICHI). https://www.who.int/classifications/ichi/en/. Accessed March 3, 2020.

[14] Li Q‐Y , Qiu Z‐Y , Chen D . Construction of national framework and data systems of functioning, disability, and health of information using ICF. Chin J Rehabil Theory Pract. 2017;23(4):385‐389.

[15] Qiu Z‐Y , Chen D . ICF based disability and rehabilitation information standard and its applications. Chin J Rehabil Theory Pract. 2014;20(6):501‐507.

[16] World Health Organization, World Bank . World Disability Report (International Chinese version) [M]. Qiu Z‐Y, trans. Geneva, Switzerland: World Health Organization; 2013.

[17] World Health. Organization . Rehabilitation in Health Service System [M]. Qiu Z‐Y, Guo J, Li L, trans. Hong Kong: Hong Kong Rehabilitation Association; 2019.

[18] Qiu Z‐Y , Li Q‐Y . World report on disability: implications to disability and rehabilitation. Disabil Res. 2012;3:9‐14.

[19] Qiu Z‐Y . World report on disability: framework, approach and implications to disability. Chin J Rehabil Theory Pract. 2013;19(10):901‐904.

[20] Qiu Z‐Y . The World Health Organization and the World Bank jointly released the first World Disability Report, an important international document on disability development. Chin J Rehabil Theory Pract. 2011;17(6):508‐511.

[21] Imamura M , Omar Z , Giraldo‐Prieto M , Lugo‐Agudelo LH . 5.5 Physical and rehabilitation medicine in health‐care systems: long‐term care and community‐based rehabilitation. J Int Soc Phys Rehabil Med. 2019;2(suppl 1):S93‐S97.

[22] Gutenbrunner C , Nugraha B . 2.1 Rehabilitation: rehabilitation as a health strategy. J Int Soc Phys Rehabil Med. 2019;2(suppl 1):S15‐S18.

[23] European Physical and Rehabilitation Medicine Bodies Alliance . White Book on Physical and Rehabilitation Medicine (PRM) in Europe. Chapter 1. Definitions and concepts of PRM. Eur J Phys Rehabil Med. 2018;54(2):156‐165. 29565102 10.23736/S1973-9087.18.05144-4

[24] European Physical and Rehabilitation Medicine Bodies Alliance . White Book on Physical and Rehabilitation Medicine (PRM) in Europe. Chapter 7. The clinical field of competence: PRM in practice. Eur J Phys Rehabil Med 2018;54(2):230‐260. 29565108 10.23736/S1973-9087.18.05151-1

[25] Wu SS , Ahn C . 3.2 Physical and rehabilitation medicine‐clinical scope: Specific health problems and impairments. J Int Soc Phys Rehabil Med. 2019;2(suppl 1):S29‐S34.

[26] Melvin JL . 2.2 Rehabilitation: rehabilitation as an intervention. J Int Soc Phys Rehabil Med. 2019;2(suppl 1):S19‐S24.

[27] Haig AJ . 3.4 Physical and rehabilitation medicine‐clinical scope: physical and rehabilitation medicine interventions. J Int Soc Phys Rehabil Med. 2019;2(suppl 1):S41‐S46.

[28] Li A , Qiu Z‐Y , Wu H . Rehabilitation 2030: international rehabilitation development and call for action. Chin J Rehabil Theory Pract. 2017;23(4):379.

[29] Li X , Qiu Z‐Y , Yang J . Rehabilitation 2030: meet ever‐increasing rehabilitation needs. Chin J Rehabil Theory Pract. 2017;23(4):380‐384.

[30] Qiu Z‐Y , Chen D . Developing health care and rehabilitation services for the health of people with disabilities ‐ learning from the WHO Global Disability Action Plan 2014–2021: improving the health of all people with disabilities. Chin J Rehabil Theory Pract. 2014;20(7):611‐615.

[31] Qiu Z‐Y , Kwok JKF , Li L . WHO rehabilitation in health system: background, framework and approach, contents and implementation. Chin J Rehabil Theory Pract. 2020;26(1):16‐20.

[32] Novel Coronavirus Pneumonia Emergency Response Epidemiology Team . The epidemiological characteristics of an out‐break of 2019 novel coronavirus diseases (COVID‐19) in China. Zhonghua Liu Xing Bing Xue Za Zhi. 2020;41(2):145‐151.32064853

[33] Liu X , Wang S , Liu L . General anatomy report of novel coronavirus pneumonia death corpse. J Forensic Med. 2020;36(2):19‐21.

[34] Wu Z , McGoogan JM . Characteristics of and important les‐ sons from the coronavirus disease 2019 (COVID‐19) outbreak in China: summary of a report of 72314 cases from the Chinese Center for Disease Control and Prevention. JAMA. 2020;323(13):1239. 10.1001/jama.2020.2648 32091533

[35] World Health Organization . Technical guidelines novel coronavirus (2019‐nCoV).https://www.who.int/zh/emergencies/diseases/novel‐coronavirus‐2019/technical‐guidance. Accessed March 10, 2020.

[36] World Health Organization . Brief Model Disability Survey.https://www.who.int/disabilities/data/mds/en/. Accessed March 10, 2020.

[37] Bickenbach J . ICF Core Classification Combination Clinical Practice Manual. Qiu Z‐Y, Li J, Wu X, trans. Beijing, China: People’s Military Medical Press; 2013.

[38] Qiu Z‐Y , Huang H‐Z , Zhang J‐M . Psychological and behavior barriers and psychological rehabilitation strategies for natural disaster survivors. Chin J Rehabil Theory Pract. 2008;14(7):673‐676.

[39] World Health Organization . Operational considerations for case management of COVID‐19 in health facility and community. https://apps.who.int/iris/bitstream/handle/10665/331492/WHO‐2019‐nCoV‐HCF_operations‐2020.1‐eng.pdf. Published March 19, 2020. Accessed March 19, 2020.

[40] National Health and Health Commission of China . Novel coronavirus infection related ICD code issued by the National Health and Health Commission of China. NO.58. http://www.nhc.gov.cn/xcs/zhengcwj/202002/dcf3333b740f4fabad5f9f908d1fc5b4.shtml. Published February 13, 2020. Accessed February 13, 2020.

[41] China Health Office Medical Service. Novel coronavirus pneumonia rehabilitation program (trial) notification. NO. 189.http://www.nhc.gov.cn/yzygj/s7653p/202003/46c9294a7dfe4cef80dc7f5912eb1989.shtml. Published March 4, 2020. Accessed March 4, 2020.

[42] World Health Organization . WHO Disability Assessment Schedule 2.0 (WHODAS 2.0). 2018;https://www.who.int/classifications/icf/more_whodas/en/. Accessed March 10, 2020.

[43] Qiu Z‐Y , Li D . Current conceptual framework and policies of disability and rehabilitation and approach of community‐based rehabilitation. Chin J Rehabil Theory Pract. 2011;17(7):601‐605.

[44] Balas MC , Vasilevskis EE , Olsen KM , et al. Effectiveness and safety of the awakening and breathing coordination, delirium monitoring/management, and early exercise/mobility bundle. Crit Care Med. 2014;42(5):1024‐1036. 24394627 10.1097/CCM.0000000000000129 PMC4105208

[45] Lun International Classification of Functionixu L , Pengming L . Respiratory Physical Therapy: On Duty Physician Manual [M]. Tianjin, China: Tianjin Science and Technology Press; 2014.

[46] Mingsheng Z , Zeguang Z , Qi G . Rehabilitation medicine series: respiratory rehabilitation [M]. Beijing, China: People’s Health Press; 2019.

[47] Rochester CL , Vogiatzis I , Holland AE , et al. An Official American Thoracic Society/European Respiratory Society Policy statement: enhancing implementation, use, and delivery of pulmonary rehabilitation. Am J Respir Crit Care Med. 2015;192(11):175‐187. 10.1164/rccm.201510-1966ST 26623686

[48] American Society of Sports Medicine . ACSM exercise testing and exercise prescription guide [M]. 10th ed Beijing, China: People’s Health Press; 2019.

[49] Bin Z , Guiying L , Mingsheng Z . Disability and rehabilitation in the elderly. Chin J Geriatr. 2019;38(10):1101‐1103.

[50] World Health Organization . Rehabilitation 2030: A Call for Action. http://www.who.int/disabilities/care/Rehab2030MeetingReport_plain_text_version.pdf. Published 2017. Accessed August 2, 2018.

